# Orthokeratology Lens Wear for 2 Years in Children Did Not Alter Tear Film Lipid Thickness by Non-Invasive Interferometry

**DOI:** 10.3389/fmed.2022.821106

**Published:** 2022-02-10

**Authors:** Haozhe Yu, Yifei Yuan, Wenyu Wu, Weizhen Zeng, Louis Tong, Yu Zhang, Yun Feng

**Affiliations:** ^1^Department of Ophthalmology, Peking University Third Hospital, Beijing, China; ^2^Singapore Eye Research Institute, Singapore, Singapore; ^3^Department of Cornea and External Diseases, Singapore National Eye Centre, Singapore, Singapore; ^4^Department of Ophthalmology, Duke-NUS Medical School, Singapore, Singapore

**Keywords:** orthokeratology, lipid layer thickness, dry eye, LipiView, meibomian gland

## Abstract

**Purpose:**

Previous studies suggest that overnight orthokeratology (OOK) may be detrimental to tear function. We aimed to investigate the effect of OOK on lipid layer thickness (LLT), blink pattern, and meibomian gland and elucidate the relationship of these variables.

**Methods:**

Thirty-seven participants who wore OOK lenses every night for at least 2 years and twenty-four healthy non-contact lens wearers (controls) were enrolled in this retrospective study. LipiView interferometry, blink pattern analysis, the ocular surface status, and morphology and function of the meibomian gland were determined.

**Results:**

The OOK group and healthy controls had similar LLT, blink patterns, ocular surface status, and the function of the meibomian gland. OOK participants demonstrated higher meiboscore in the lower eyelids (*p* < 0.05) but not in the upper eyelids. Within the OOK group, LLT was significantly impacted by the partial blink rate (*p* < 0.05) and the total score of the upper meibomian gland (*p* = 0.10).

**Conclusions:**

Wearing OOK for 2 or more years increased lower eyelid meibomian gland dropout but did not have a reduction of LLT.

## Introduction

With the widespread availability of electronic devices, the prevalence of myopia in children is rising rapidly and is considered a major public health issue worldwide, especially in East Asia (1). It has been reported that 80% of high school graduates in China suffer from myopia, which greatly increases the incidence of blinding diseases like glaucoma and macula lutea, resulting in a disease burden of over $240 billion per year (2, 3). Several methods of preventing myopia have been proposed, of which Overnight Orthokeratology (OOK) has been proved to be one of the most effective and widely used options in recent years (4, 5). Through reshaping the corneal epithelial tissue to change refractive and present myopic defocus in the peripheral retina, OOK can effectively slow down the elongation of the axial length by +0.70 mm after a 2 year treatment (6, 7).

Several studies have reported on the potential impact of OOK on diseases of the ocular surface, including corneal staining and infectious keratitis (8). OOK wearers may suffer from contact lens-related dry eye, which manifests as increased tear evaporation rate, reduced tear film thickness, and increased frequency of incomplete blinks. Such dry eye problems are common reasons for the discontinuation of OOK-wear (9). It is generally accepted that these tear film complications might be attributed to meibomian gland dysfunction (MGD) instead of hypoxia given the high oxygen permeability design of the OOK (10, 11).

Despite several reports that explored the association between OOK and the structure and function of the meibomian gland in OOK-wears (12, 13), these relationships remain controversial because lipid layer analysis was rarely performed and the effect of long term OOK has not been sufficiently evaluated. Recently, LipiView interferometry, which can accurately determine tear lipid layer thickness and rate of incomplete blinks, has become available (14–16). This offers an opportunity to re-evaluate the relationship between OOK and MGD, along with other tear film parameters.

The main purpose of this study was to evaluate the changes in the meibomian gland, lipid layer, and incomplete blinking in OOK-wears based on LipiView to reveal the potential etiology of OOK-related dry eye.

## Methods

### Design and Patients

This retrospective study was conducted at the Department of Ophthalmology, Peking University Third Hospital from January 2019 to September 2019. The inclusion criteria were as follows: (1) Age > 10 years; (2) myopia from −1.0D to −5.5 DS, and with-the-rule astigmatism of up to −1.75 DC or against-the-rule astigmatism of < -0.75 DC with keratometry from 41 to 46 D; (3) For the OOK group, patients should have been able to master the wearing of OOK lenses and had worn them for 2 years; and (4) for the control group, all patients wore no glasses or used mainly frames, rarely using contact lenses (<2 h/d and <2 d/1w). Consent of guardians was obtained for minors who met the aforementioned inclusion criteria. The exclusion criteria were patients who were previously diagnosed with dry eye disease (DED), had a daily screen time ≥ 2 h, had pathological changes of the lid margin or cornea, uvea, or retina, had other systemic diseases that may influence the ocular surface, and received other ocular treatment or surgeries.

The sample size was estimated through PASS software (NCSS, LLC., USA) with LLT as the primary outcome under the power of the test (1–β) = 0.8 and significance level (α) = 0.05. The difference in LLT between the OOK and control groups was set to be significant at 10 ± 15 mm according to previous research (16–18). The 1:1 equal sample size design was used in this study, and the sample size for each group was calculated to be 37. The study was approved by the Institutional Ethics Committee of Peking University Third Hospital (M2018171), and all procedures were conducted in accordance with the principles of the Declaration of Helsinki.

### Ocular Examination

All subjects were asked to avoid using any eye cream and ocular medications for at least 24 h prior to the clinical measurements, and for the OOK group, contact lenses should be removed more than 4 h in advance. The ocular surface disease index (OSDI) was conducted to compare the DED-related symptoms between OOK and healthy control, and the total scores of 13, 23, and 33 represented the cut points of mild, moderate, and severe DED symptoms, respectively (19). The cornea staining and tear break-up time (TBUT) were conducted through a fluorescein strip (Meizilin Co., Ltd. China) to evaluate the ocular surface status and tear film stability. The score of cornea staining followed by the Oxford grading scheme and the TBUT <10 s was considered consistent with the diagnosis of DED (20). For tear secretion evaluation, the Schirmer test sterile strips (Meizilin Co., Ltd. China) were inserted into the lower conjunctival sac, the wetting paper was measured after 5 min with the eye closed, and the wetting length > 10 mm were considered to be normal (21). The physiological function of the meibomian gland was assessed by observing the meibomian gland expressibility and its properties in the central five meibomian glands of the eyelids. Zero referred to all meibomian glands secreting normally or the meibum presenting as clear and transparent, while 3 stood for none of the meibomian gland secreting meibum or the meibum presenting toothpaste-like consistency (22). The total score of the meibomian gland was defined as the sum of meiboscore, meibomian gland expressibility, and its properties score.

### LipiView Measurement

During the examination, the patients were asked to look at the front light-spot to ensure their pupils were directly in the center of the interferometer camera with natural blinking, and all the measurements were conducted by the same experienced examiner using LipiView Ocular Surface Interferometer (Johnson & Johnson, USA). The average lipid layer thickness (LLT), partial blinks and their rate (PBR, per 20s), and complete blinks of subjects were recorded, and the patients with an outcome conformance factor (CF) of <0.8 were asked to repeat the measurement. The morphology of the upper and lower eyelid meibomian gland in these subjects were observed using the photographic module of LipiView, and the extent of meibomian gland loss was scored as follows (meiboscore): 0 (no loss), 1 (loss area <33%), 2 (34% < loss area <66%), and 3 (loss area >67%) (23).

### Statistical Method

Statistical analysis was performed by IBM SPSS Statistics 24.0 (IBM SPSS Inc., USA) and R software 4.0.4, and the measurement data subjected to normal distribution were presented as the mean ± SD (M ± SD) while the enumeration data were presented as the ratio (%). Their differences between the groups were compared through *t*-test (demographic data), linear mixed models (ophthalmic examination data), and Clustered Wilcoxon rank sum test (enumeration data), respectively (24, 25). The generalized estimating equation was used to assess the relationship among various factors and LLT (26). The significant level was set up as α = 0.05.

## Results

A total of sixty-one participants (122 eyes) were recruited into the study and completed the LipiView examination, which met sample size requirements. The OOK group consisted of thirty-seven cases (74 eyes) who had worn OOK lenses for 2 years with an average age year of 12.7 ± 1.9 and male/female = 20:17. Twenty-four cases (48 eyes) who were without a history of contact lens wearing and dry eye with an average age year of 12.9±7.7 and male/female = 13:11 were included in the control group. There was no statistical difference between each group in age (*p* = 0.92) and sex (*p* = 0.99). Additionally, the CF in both the OOK group (0.94 ± 0.06) and control group (0.93 ± 0.08) were higher than 0.8, indicating the high accuracy and reliability of the examination.

### Intergroup Comparison of the Ocular Surface Examination

**Figure 1:**
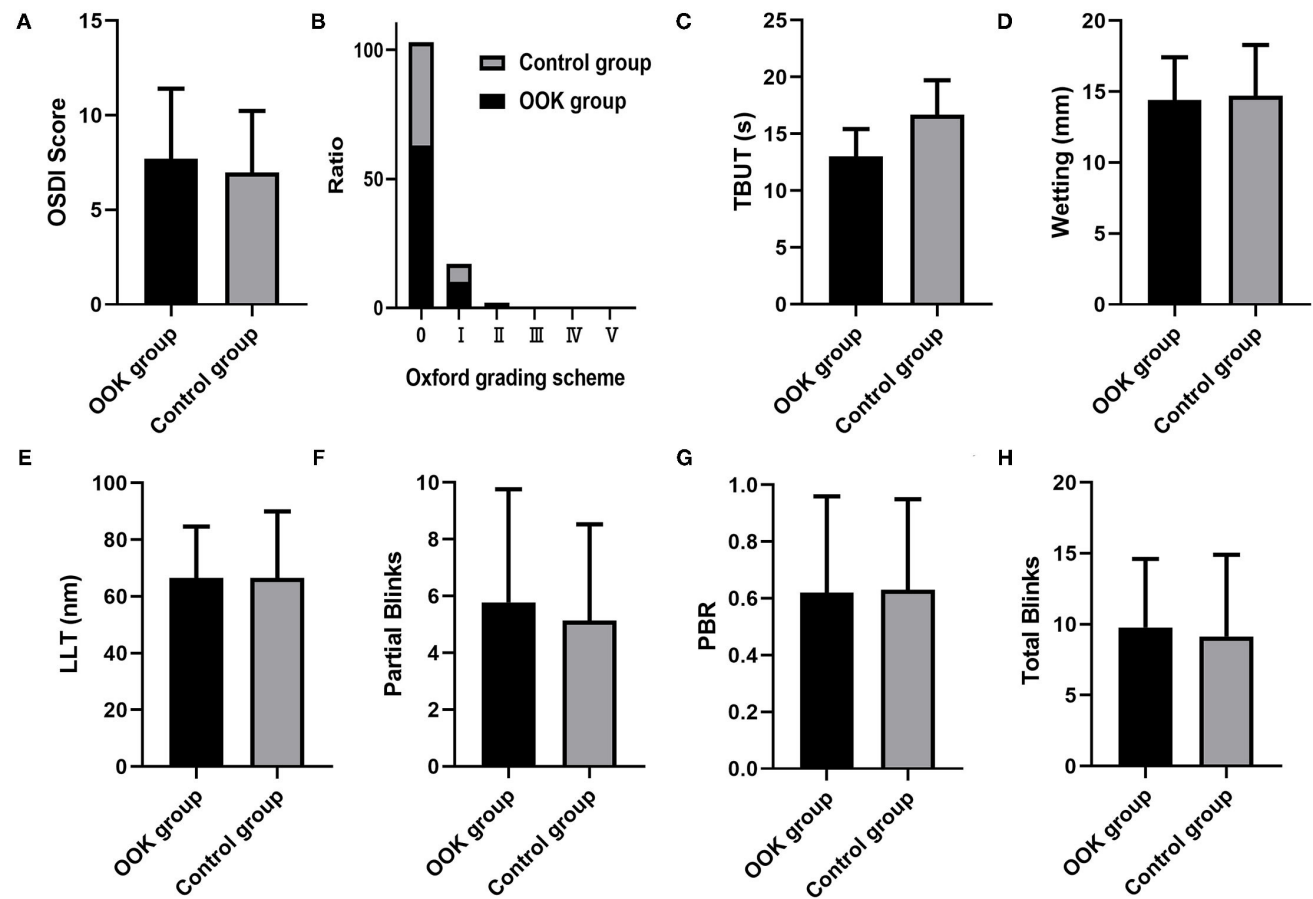
Comparison of **(A)** ocular surface disease index (OSDI) score, **(B)** cornea staining score, **(C)** tear break-up time (TBUT), **(D)** tear secretion, **(E)** LLT, **(F)** partial blinks, **(G)** partial blinks and their rate (PBR), and **(H)** total blinks between orthokeratology (OOK) group and control group.

The results of the ocular surface examination and LipiView examination were summarized in Figure 1. There was no difference between OOK group and control group in OSDI (7.69 ± 3.71 vs. 6.98 ± 3.25, *p* = 0.44), cornea staining (score = 0: 85.1 vs. 83.4%, *p* = 0.82), TBUT (13.03 ± 2.40 vs. 13.67 ± 3.05, *p* = 0.36), Schirmer test (14.40 ± 3.02 vs. 14.71 ± 3.58, *p* = 0.71), LLT (66.53 ± 18.12 vs. 66.42 ± 23.52, *p* = 0.77), partial blinks (5.78 ± 3.98 vs. 5.14 ± 3.38, *p* = 0.44), PBR (0.62 ± 0.34 vs. 0.63 ± 0.32, *p* = 0.91), and total blinks (9.78 ± 4.83 vs. 9.14 ± 5.77, *p* = 0.54).

### Comparison of Morphology and Function of Meibomian Gland

**Figure 2:**
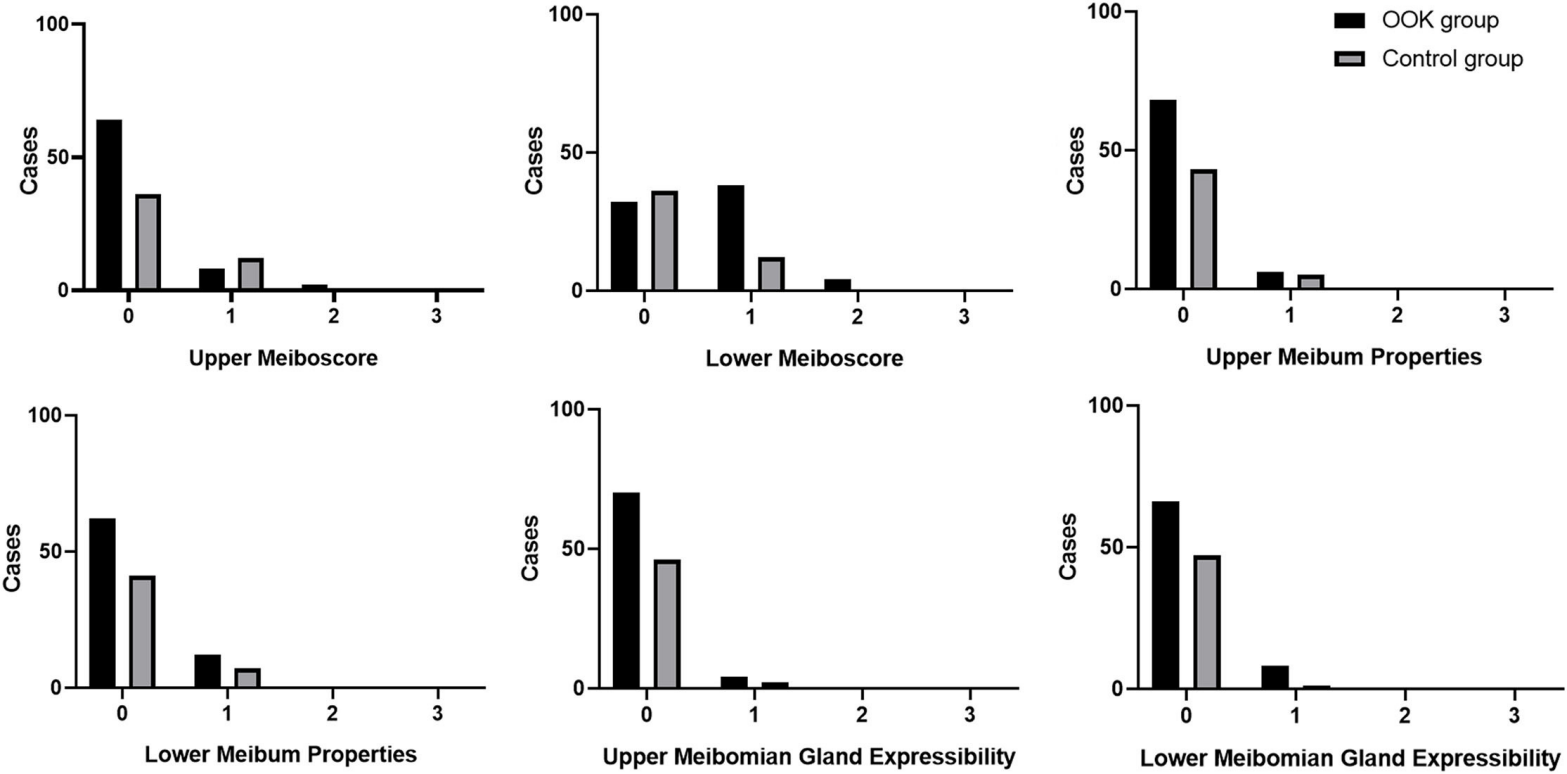
Meiboscore, meibum properties, and meibomian gland expressibility of the upper and lower eyelid in the OOK group and control group.

Figure 2 demonstrated the distribution of meibomian gland dropout in the OOK group and control group, and the meiboscore of each group was lower than 3. In the lower eyelids, the OOK group showed a higher proportion of light (51.35%) and medium (5.40%) loss of meibomain glands, while the meiboscore was limited to mild loss (1) without any case of moderate or severe loss in the control group (33.3%) (*p* < 0.01). There was no statistically significant difference in the upper meibomian gland dropout (*p* = 0.25), upper and lower score of meibum properties (*p* = 0.71, 0.83), and meibomian gland expressibility (*p* = 0.79, 0.13) in each group.

### Correlation Between LLT and Other Factors

The generalized estimating equation model was established to evaluate the associations of LLT with demographic factors, blink-related parameters, and the total score of the meibomian gland (Table 1). The LLT was significantly affected by the total score of the meibomian gland as well as PBR in the control and OOK groups, respectively. In addition, it could be considered that the lower total score of the upper meibomian gland was related to higher LLT in the OOL group with a significance level of 0.10.

**Table 1:** Associations of lipid layer thickness (LLT) and other parameters in generalized estimating equations models.

**Variable**	**OOK group**		**Control group**	
	***B***	***P***	***B***	***P***
Age	−0.90	0.24	−0.56	0.45
Gender	0.91	0.83	0.11	0.74
Years of lens	3.88	0.94	–	–
Partial Blink	0.34	0.61	0.34	0.76
PBR	17.30	*P* < 0.001	3.24	0.76
Total blink	−0.50	0.13	0.91	0.08
Total score of upper meibomian gland^*^	−2.30	0.10	−11.37	*P* < 0.05
Total score of lower meibomian gland^*^	−1.43	0.25	−7.25	*P* < 0.05

* *Compared with score = 0*.

## Discussion

In this study, we found LLT to be unaffected by OOK-wear and lower eyelid meibomian gland atrophy to be increased.

The lipid layer located in the superficial layer of the tear film is mainly formed by meibomian lipids secreted from the upper and lower meibomian gland (27, 28). The lipid layer was associated with contact lens-related ocular dryness and discomfort because an abnormal lipid layer could result in a high tear evaporation rate, low tear breakup time, and high tear osmolarity value (29–31). The LLT measured by LipiView interferometry is considered an important indicator of tear lipid health and demonstrates a good correlation with meibomian gland function (32–34). In this study, we do not observe any effect of long-term wearing of OOK on the LLT and the ocular surface status which is consistent with previous reports of high comfort levels in OOK wearers (11, 35, 36).

Previous studies of OOK wearers have reported changes in meibomian gland morphology, tear cytokines, and ocular surface parameters (13, 35).

The meibomian gland plays a vital role in maintaining the stability of tear film, and an increased meiboscore is generally considered as the important risk factor for DED and contact lens-related discomfort (37, 38). Previous studies have examined the influence of OOK wearing on the morphology of the meibomian gland based on non-contact infrared meibography or Keratograph 5M and found that there is no significant difference in the meiboscore before and after wearing lenses (12, 39). The discrepancy might be attributed to the imaging quality of different instruments. Wong et al. (40) found that the LipiView was easier in evaluating the extent of the meibomian gland, owing to its high contrast and low reflectivity compared with Keratograph 5M. Alternatively, the duration of OOK lens wear may be shorter in previous studies and so, insufficient to cause meibomian gland dropout.

Mechanical trauma and inflammation are the main hypotheses in explaining the contact lens-related meibomian gland damage. Overnight lens wearing plus rapid-eye-movement might further prolong the eyelid/lens contact duration and increase friction between the lens and the meibomian gland (41). Pult et al. (42) and Eom et al. (43) found the destruction of the meibomian gland in MGD and DED patients to be more severe in the lower eyelid. The upper eyelid could be relaxed to a larger extent to compensate for the damage induced by OOK (43–45). It may be that the lower eyelid is more irregular (46), and so predisposed to greater meibomian gland dropout. Considering that the upper eyelid usually shows a larger area lens than the lower eyelid during sleep, its deformation might be less affected by wearing OOK lenses, which results in a more regular lid margin and lower meiboscore.

It is generally accepted that the duration of contact lens plays a more critical role on the meibomian gland, rather than lens material (47). If anything, however, OOK lens materials have higher oxygen permeability and induce less hypoxia and inflammation (48–50). In addition, the MGD was also considered to be posterior blepharitis involving various inflammatory processes. The major reason that there was no difference in physiological functions of the meibomian gland between OOK and healthy control might be attributed to such characteristics of OOK (51, 52).

Blink can maintain tear film stability of the ocular surface by promoting the reconstruction and development of the lipid layer (53, 54). can be covered by tears through complete blinking, preventing damage from DED. Therefore, partial blinking is deleterious (55). Several studies have reported increased partial blinks in contact lens wearers, which have also been reported in DED (56, 57). However, we did not show OOK affecting PBR, likely due to underpowering for this variable.

As seen in previous studies, there was an exact correlation of LLT with the total score of the meibomian gland in the healthy control from the regression analysis results, which could be attributed to how the meibomian gland secreted lipid layer and stabilized them (58). In the OOK group, the PBR demonstrated a significant positive effect on LLT. Nevertheless, the relationship between partial blink and LLT was still unclear because of its complex influencing factors from meibomian gland function, tear composition, and flow rate (27). Li et al. (59) reported that the LLT showed significant negative correlations with incomplete blinking rate, while Jie et al. (60) presented that there was not an association between them. In this study, a reason proposed was that the upper meibomian gland could produce a stable tear film alone after a partial blink and the lower meibomian gland did not contribute to it, while the LLT was essential for tear film stability (61, 62). Based on this, a partial blink was considered a compensatory response to an abnormal tear film (63). It can be speculated that the function of the upper and lower meibomian gland might be reconfigured after a long time of wearing OOK lenses, and the PBR became a protective factor of LLT and tear film in OOK wearers. Furthermore, the significance of the total score of the upper meibomian gland in regression analysis indicated that the LLT was more likely to be influenced by the upper eyelid than the lower, which further supported this hypothesis.

There are a few limitations to our study. This is single-center research, and the limited number of participants enrolled in this study might increase potential sampling bias. Moreover, there was a lack of longitudinal assessment about the effect of long-term wearing OOK on the ocular surface, LLT, blink-related parameters, and the meibomian gland. Future studies are needed to evaluate tear composition and distribution, and how these are influenced by LLT, PBR, and meibomian gland status.

## Data Availability Statement

The raw data supporting the conclusions of this article will be made available by the authors, without undue reservation.

## Ethics Statement

The studies involving human participants were reviewed and approved by Peking University Third Hospital Medical Science Research Ethics Committee. Written informed consent to participate in this study was provided by the participants' legal guardian/next of kin.

## Author Contributions

YZ and YF designed the study. HY and YY wrote the initial draft. YY, WW, WZ, LT, and YF revised the manuscript. All authors made a substantial and intellectual contribution to the article and approved the submitted version.

## Funding

This study was supported by the National Natural Science Foundation of China grants (Nos. 81700799 and 82070926).

## Conflict of Interest

The authors declare that the research was conducted in the absence of any commercial or financial relationships that could be construed as a potential conflict of interest.

## Publisher's Note

All claims expressed in this article are solely those of the authors and do not necessarily represent those of their affiliated organizations, or those of the publisher, the editors and the reviewers. Any product that may be evaluated in this article, or claim that may be made by its manufacturer, is not guaranteed or endorsed by the publisher.
